# Mesenchymal Stem Cells: A Hope or a Hype?

**DOI:** 10.3390/ijms241713218

**Published:** 2023-08-25

**Authors:** Heba Abdelrazik

**Affiliations:** Galliera Hospital, Genoa Italy, Cairo University, Cairo 12163, Egypt; hebatalla.abdelrazek@galliera.it; Tel.: +39-348-793-7713

Mesenchymal Stem Cells (MSC) represent a captivating field of research attempting to address the vast variety of disease burdens, which at present lack efficient treatment. Although more than 3500 MSC clinical trials are currently ongoing with almost 10,000 trials already registered on “www.clinicaltrials.gov (accessed on 10 July 2023)”, they do not fulfill their promises, as MSC are not yet in routine clinical practice.

Thus, the objective of this Special Issue on MSC biology was to provide a venue for novel research articles and reviews that update and gather the current knowledge on the progress in this research topic. Herein is an overview of what to expect from the first Special Issue.

Initially, MSC were mainly ***derived*** from bone marrow (BM), cord blood (CB), Wharton Jelly (WJ), and adipose tissue (AT), while at present MSC can be isolated from almost all organs and tissues [1]. Articles in this issue isolated MSC from the tonsils, the heart, from benign tumors, such as as lipomas to induce wound healing, as well as from the tumor microenvironment. Genetically engineered MSC for protein-coding and non-coding transcripts were reviewed to enhance their pro-regenerative properties and accelerate bone healing.

Initially, MSC gained attention due to their ***immunomodulatory*** [2] and ***regenerative*** capacities; in this Special Issue, a review and a couple of research articles therefore addressed the current situation of the use of MSC in adipogenesis, osteoarthritis, tendon regeneration, and bone healing. Later, MSC were considered as the keepers of ***tissue homeostasis*** [3], having anti-oxidative and angiogenic activity, of which a review is herein included. MSC are deeply involved in ***neurobiology*** due to their neuroprotective effects; they support neuron survival, axonal growth, and control of glial scarring. Thus, their use in spinal cord injuries was also reviewed. Interesting, and among the novel methodologies described herein, is the differentiation technique of tonsil-derived MSC into motor-neuron-like cells that secrete acetylcholine and their possible development into neuromuscular junction formations. Work has been carried out on thrombin-preconditioned WJ-MSC, which attenuated severe hypoxic-ischemic-encephalopathy-induced brain infarction and improved behavioral function tests in rats. Moreover, novel signals involved in the mesenchyme molecular cross talks in ***embryogenesis*** have been discovered.

The therapeutic effects of MSC involve most of the body’s organs, whether healthy or diseased [4], in adults or children, and pre-natal therapeutic advances have even been made.

Another aspect was unraveling of some of the ***regulatory mechanisms*** of MSC, such as the role of low-intensity pulsed ultrasound (LIPUS) in intra and extracellular cytoskeletal remodeling. Advancement in molecular techniques together with engaging MSC research in ***bioinformatic*** analysis underlines the role of micro RNA to expand the clinical potential of MSC.

A review article discussed the discoveries of how those cells can remotely manipulate different diseases, including the tumor ***microenvironment***, which is currently emerging as a key player in promoting drug resistance and overcoming the cytotoxic effects of drugs. A communication article referred to the role of Cyclophilin A in cardiac-derived MSC, paving the way to their future implications in ***cardiac remodeling*** in arrhythmogenic cardiomyopathy.

Concerns about possible side effects of cellular differentiation and ***tumorigenesis*** have hindered the translation of cellular therapeutic advances [5]. For this purpose, a review addressed the genetic stability of MSC for biosafety. Although the senescence of cultured MSC would be considered safer, yet may lower MSC efficacy, two contributionsdissected and modulated MSC senescence in two different in vivo models, i.e., mouse and zebrafish embryo extract models, respectively.

Additional articles addressed the manipulations of MSC conditioned media to utilize their paracrine properties and cellular preconditioning to guide MSC towards selected pathways/lineages, including tendon cells. They showed that changing the cellular environment can modify the properties of MSC and prompt safer therapeutic approaches. Another alternative to cell therapy, ***MSC vesicles*** opened a gateway to cell-free therapy, as has been reviewed herein for corneal diseases utilizing genetically modified vesicles, or in the case of enhanced vesicle biogenesis boosted by thrombin-mediated preconditioning of CB-MSC.

Altogether, the articles published in this Special Issue raise more questions than they answer, given that most of the conclusions carry the statement ‘***further studies are needed’***. This indicates that basic research is fundamental and is needed to evaluate all the open questions that remain. A better relationship between basic/animal studies and clinical trials with a back-and-forth approach could be the key to translating fascinating promises realities for patients. Thus, there is a need for more studies dedicated to MSC and also targeting specific goals and unmet needs, further bridging basic science and clinical efforts in cell therapy and regenerative medicine.
